# Impacts of Long-Term High-Temperature and Low-Salinity Stress on the Circadian Rhythms of Antioxidant, Immune, and Endocrine Systems in Turbot (*Scophthalmus maximus*)

**DOI:** 10.3390/antiox15020257

**Published:** 2026-02-17

**Authors:** Zhifeng Liu, Mingchao Yang, Yuelei Shi, Yilin Wang, Junlian Zhong, Yunyi Gao, Aijun Ma

**Affiliations:** 1 State Key Laboratory of Mariculture Biobreeding and Sustainable Goods, China-ASEAN Belt and Road Joint Laboratory on Mariculture Technology (Qingdao), Qingdao Key Laboratory for Marine Fish Breeding and Biotechnology, Yellow Sea Fisheries Research Institute, Chinese Academy of Fishery Sciences, Qingdao 266071, China; liuzf@ysfri.ac.cn (Z.L.);; 2 College of Fisheries and Life Science, Shanghai Ocean University, Shanghai 201306, China; 3 Fisheries College, Zhejiang Ocean University, Zhoushan 316022, China

**Keywords:** *Scophthalmus maximus*, chronic stress, antioxidant enzymes, non-specific immune, endocrine, circadian rhythm

## Abstract

Turbot (*Scophthalmus maximus*) is an economically vital cold-water fish frequently challenged by summer heat and low salinity. However, the temporal response of physiological circadian rhythms to such long-term stress remains underexplored. This study investigated antioxidant, immune, and endocrine rhythms in turbot acclimated to control (16 °C, 30 ppt), high-temperature (23 °C), and low-salinity (10 ppt) conditions for 30 days. Subsequently, time-series sampling was performed every 4 h for 72 consecutive hours. Under optimal conditions, hepatic superoxide dismutase (SOD), serum alanine aminotransferase (ALT), and melatonin exhibited robust 24 h rhythms. Long-term stress disrupted this homeostasis through divergent mechanisms. Low-salinity stress induced “rhythmic remodeling,” maintaining balance via phase shifts or novel infradian (48–72 h) oscillations in thyroid hormones (T3, T4) and ALT, without oxidative damage. Conversely, high-temperature stress triggered “rhythmic collapse,” characterized by a loss of daily rhythms in SOD and ALT, sustained inflammation indicated by elevated acid phosphatase (ACP), metabolic depression (suppressed T3), and malondialdehyde accumulation. These findings demonstrate that heat stress poses a more destructive threat to circadian integrity than hyposmotic stress. Consequently, the rhythmic dynamics of ACP, ALT, T3, and T4 are identified as critical indicators of stress status, serving as potential biomarkers for screening stress-tolerant strains for selective breeding.

## 1. Introduction

Circadian rhythms are intrinsic mechanisms by which organisms adapt to the alternation of day and night caused by the Earth’s rotation, regulating almost all major physiological functions, including behavioral activity, metabolism, growth, and endocrine processes [1]. These rhythms are present not only in higher animals, such as mammals and birds, but also widely distributed among invertebrates, plants, and unicellular organisms [2,3]. Although circadian rhythms have been extensively studied in terrestrial animals, their importance in aquatic animals has also gained increasing attention in recent years. Aquatic animals such as fish similarly rely on circadian rhythms to regulate daily physiological and behavioral activities, such as feeding, swimming, and reproduction [4,5].

The normal functioning of circadian rhythms is influenced by various aquaculture environmental factors, such as light, temperature, salinity, dissolved oxygen, and stocking density [6,7]. These factors not only affect the formation of circadian rhythms but can also alter fish metabolism and behavior by disrupting physiological homeostasis. For example, changes in photoperiod can directly affect fish feeding behavior and metabolic rate, whereas changes in water temperature may lead to endocrine imbalance, thereby affecting growth rate and reproductive capacity [8]. When fish face new environmental stress, their circadian homeostasis often changes, leading to short-term physiological disturbances. For example, when confronted with abrupt changes in temperature or salinity, the endocrine system of fish rapidly adjusts the secretion of cortisol and other stress hormones to respond to the external changes, which often disrupts multiple physiological rhythms such as digestion, immunity, and metabolism [9,10]. Although environmental stress can cause temporary circadian imbalance, fish usually readjust their physiological rhythms through adaptive changes and establish a new stable state suited to the new environment. Fish exposed to moderate environmental stress can reshape their rhythmic homeostasis by adjusting their internal biological clock, thereby enhancing their adaptability to the new conditions; however, if environmental stress exceeds their tolerance limits, the organism will be unable to maintain a normal rhythm cycle, and physiological rhythms may collapse. In extreme cases, this imbalance can threaten fish health and even lead to death [11]. Therefore, by monitoring changes in the circadian homeostasis in populations or families, their environmental tolerance can be effectively assessed, providing a scientific basis for healthy aquaculture and the genetic breeding of stress-resistant traits. However, research on the effects of different farming environments on circadian rhythms is relatively scarce, and most studies have focused on short-term changes in environmental factors or on non-stress factors, such as photoperiod and feeding strategies in long-term aquaculture systems. For example, existing studies have examined the effects of different feeding methods on the digestive enzymes of the flathead mullet (*Mugil cephalus*) [12] and the regulation of daily stress rhythms in the pikeperch (*Sander lucioperca*) by changes in the light spectrum [13]. Nevertheless, studies on the adaptability of circadian rhythms under long-term environmental stress remain relatively scarce.

The circadian rhythms of antioxidant systems, innate immune systems, and the endocrine system play crucial roles in fish, not only affecting their defense responses to oxidative stress but also regulating their metabolism and behavior, with profound impacts on health and growth [14,15,16]. The assessment of fish antioxidant, immune, and endocrine rhythms is usually achieved by monitoring diurnal fluctuations of a multi-dimensional panel of markers: antioxidant enzymes (e.g., total antioxidant capacity T-AOC, superoxide dismutase SOD, catalase CAT, glutathione peroxidase GSH-Px, malondialdehyde MDA) to reflect oxidative defense rhythms; non-specific immune indices (e.g., acid phosphatase ACP, total protein TP, alanine aminotransferase ALT, aspartate aminotransferase AST) to indicate health status; and endocrine hormones (thyroid hormones triiodothyronine T3, thyroxine T4, melatonin MT, norepinephrine NA) to reveal metabolic and circadian regulation [17,18,19,20,21,22]. Although some studies have revealed circadian variation patterns and environmental regulatory effects for certain physiological and biochemical indices in fish, most related studies have focused on model species or short-term stress experiments [23,24,25], and systematic studies of physiological rhythms in economically important fish under long-term aquaculture conditions remain relatively scarce. Many previous studies have employed acute stimuli or single-factor interventions (for example, instantaneous changes in lighting, abrupt temperature shifts, or short-term salinity stress) to observe changes in hormones or metabolites of fish at different times of day [26]. However, these conditions do not fully replicate the environments to which farmed fish are chronically exposed in production. In the model species zebrafish (*Danio rerio*), it has been found that acute stress treatments can disrupt the circadian secretion rhythms of stress hormones (for example, diurnal cortisol) [27,28]; however, in contrast to these well-documented acute responses [28,29], data on how physiological circadian rhythms change in farmed fish under actual production conditions (such as sustained high-temperature or low-salinity conditions) remain relatively scarce.

*Scophthalmus maximus* (commonly known as turbot) is a flatfish of the family Scophthalmidae with high economic value in China’s marine aquaculture industry. In recent years, with the expansion of farming scale and regions, turbot culture has gradually extended from northern factory-based hatcheries to southern coastal areas (a north–south relay farming pattern), and attempts have been made to utilize tidal ponds and other environments for cultivation. Under these new farming regimes, turbot are often exposed to summer water temperatures that exceed their optimal growth temperature and to water salinity levels lower than normal seawater, thereby facing considerable environmental stress [29]. Therefore, improving the tolerance and adaptability of turbot to adverse conditions such as high temperature and low salinity has become a major focus in aquaculture production; some studies have begun to breed heat-tolerant turbot strains through selective breeding. Based on the above background, we formulated the following hypothesis: Since temperature acts as a controlling factor that globally dictates metabolic rates and enzyme stability, while salinity primarily imposes an osmoregulatory energetic load, we hypothesized that the circadian system of turbot would respond divergently to these stressors. To test this hypothesis, this study selected 16 °C and 30 ppt as the control group (simulating the optimal farming conditions for turbot), 23 °C and 30 ppt salinity as the high-temperature group (simulating summer high-temperature water), and 16 °C and 10‰ salinity as the low-salinity group (simulating low-salinity pond farming conditions), in order to comparatively analyze differences in circadian physiological indices of turbot under different temperature and salinity conditions and to explore the day-night fluctuations of immune and endocrine-related rhythmic indicators under these different environments. The aim of this study was to systematically monitor the diurnal fluctuations of downstream physiological outputs (immune, antioxidant, and endocrine rhythms) in turbot under these conditions, rather than the molecular core clock mechanism itself. By validating these distinct rhythmic responses, we aim to provide a theoretical basis for the concepts of “rhythmic collapse” versus “remodeling” in aquatic stress biology. The expected results aim to reveal the effects of environmental changes on the immune and endocrine rhythms of turbot, providing a basis for the scientific evaluation of fish environmental adaptability and theoretical support for the healthy farming and selective breeding of stress-resistant traits in turbot.

## 2. Materials and Methods

### 2.1. Ethics Statement

All experimental procedures were conducted in strict compliance with the guidelines established by the Institutional Animal Care and Use Committee of the Yellow Sea Fisheries Research Institute (Qingdao, China; approval number: YSFRI-2023014).

### 2.2. Experimental Fish and Rearing Management

Experimental fish were obtained from Shandong Weihai Guangyuhan Marine Biotechnology Co., Ltd. (Weihai, China). One hundred female and 60 male fish were selected for artificial fertilization. After the F1 generation reached 5 months of age, 900 juvenile turbot with active feeding behavior and no external injuries were randomly selected and transported to the Langya Base (Huangdao) of the Chinese Academy of Fishery Sciences for rearing. The average body weight was 24.81 ± 3.58 g, and the average body length was 9.25 ± 0.56 cm. The experimental fish were reared for 2 weeks in cylindrical white plastic buckets (diameter 1.2 m, height 1.5 m, volume 1.6 m^3^; 9 buckets, 100 fish per bucket; the stocking density was approximately 90 fish/m^2^, reflecting commercial aquaculture conditions [30]) at 16 °C, 30 ppt, and dissolved oxygen > 7 mg/L. During the second week of acclimation, fish were fed twice daily with commercial feed.

### 2.3. Experimental Design and Sampling

Three experimental groups were established with different culture conditions: the high-temperature group (23 °C, salinity 30 ppt), the low-salinity group (16 °C, salinity 10 ppt), and the control group (16 °C, salinity 30 ppt). Each group had three white plastic buckets for rearing. After acclimation, the high-temperature group was increased by 1 °C per day for 7 days to reach 23 °C, and the low-salinity group was decreased by 3 ppt per day for 7 days to reach 10 ppt. Water temperature was controlled by a chiller, and salinity was adjusted by mixing seawater and fresh water. The photoperiod was controlled automatically (light from 06:00 to 20:00, dark from 20:00 to 06:00). Fish were reared in a flow-through system with continuous water renewal, which effectively prevented the accumulation of metabolic wastes and maintained optimal and stable water quality conditions throughout the experiment (pH 7.61–7.82, dissolved oxygen > 7 mg/L, and ammonia nitrogen < 0.14 mg/L). Fish were fed commercial feed twice daily (06:00 and 18:00) to satiation, and uneaten feed and feces were promptly removed to maintain water quality. The rearing period was 30 days. After the 30-day rearing period, samples were taken every 4 h for 72 h (at 08:00, 12:00, 16:00, 20:00, 24:00, and 04:00). At each sampling time, 9 fish from each group were randomly selected. Since juvenile turbot at this stage lack external sexual dimorphism and the population sex ratio is approximately 1:1, a random sampling strategy was employed to minimize potential sex-dependent bias. Fish were anesthetized with 200 mg/L MS-222, and 0.5 mL of tail vein blood was collected into a 1.5 mL centrifuge tube. After blood collection, the fish were immediately dissected, and liver tissue was collected. Liver samples were snap-frozen in liquid nitrogen and stored at −80 °C. The collected blood samples were held at 4 °C for 6–8 h, then centrifuged at 4 °C (3000 rpm, 10 min). The serum was separated and stored at −80 °C for further analysis.

### 2.4. Measurement of Liver Antioxidant Indices

Liver antioxidant indices included T-AOC, SOD, CAT, GSH-Px, and MDA. All were measured using commercial kits from Nanjing Jiancheng Bioengineering Institute (Nanjing, China). The specific methods and kit numbers were: T-AOC—measured by the microplate method (kit no.: A015-2-1); SOD—measured by the WST-1 method (kit no.: A001-3); CAT—measured by the ammonium molybdate method (kit no.: A007-1-1); GSH-Px—measured by the colorimetric method (kit no.: A005-1); and MDA—measured by the TBA method (kit no.: A003-1). All measurements were performed strictly according to the kit instructions.

### 2.5. Measurement of Serum Non-Specific Immune and Endocrine Indices

Serum non-specific immune indices included ALT, AST, ACP, and TP. ALT and AST were measured by the microplate method, ACP by the micro-enzymatic method, and TP by the BCA microplate method. Kits from Nanjing Jiancheng Bioengineering Institute were used (ALT kit no.: C009-2-1; AST kit no.: C010-2-1; ACP kit no.: A060-2; TP kit no.: A045-4). All procedures followed the kit protocols.

Endocrine indices included T3, T4, MT, and NA. T3 and T4 were measured by enzyme-linked immunosorbent assay (ELISA), and MT and NA were measured by enzyme immunoassay. T3 and T4 kits (YP-90019 and YP-90004) were from Beijing I-Lai Rui Biotechnology Co., Ltd. (Beijing, China), and MT and NA kits (YS-X41020 and YS-Y10028) were from Shanghai Yushao Biotechnology Co., Ltd. (Shanghai, China). All steps were performed according to the respective kit instructions.

### 2.6. Data Processing and Analysis

Experimental data were organized in Excel 2016 and expressed as mean ± standard deviation. Statistical analysis: Independent-sample *t*-tests (SPSS 18.0) were used to examine differences between groups (low salinity vs. control, high temperature vs. control) for both overall and specific time points. Significance was set at *p* < 0.05. Rhythmicity analysis: Cosine analysis was performed using an online Cosine tool to determine significant rhythms for each index (https://cosinor.online/app/cosinor.php, accessed on 6 August 2025). This time-series analysis, based on a least-squares model, evaluated the statistical significance of fluctuations with a significance level of *p* < 0.05.

## 3. Results

### 3.1. Effects of Different Rearing Environments on Antioxidant Enzymes in Juvenile Turbot Liver

**Figure 1:**
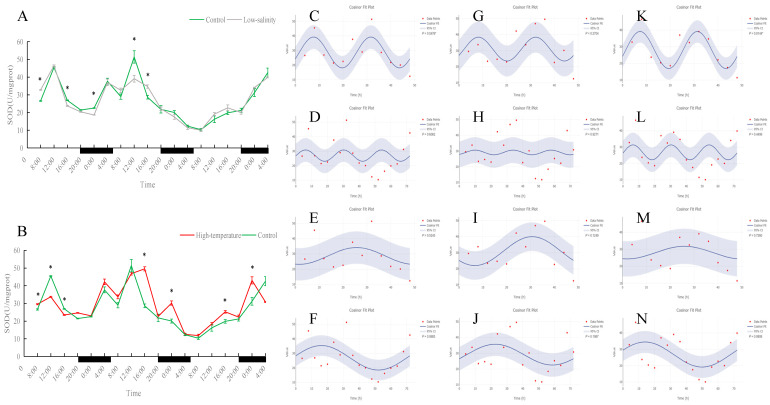
Temporal variations and rhythmicity analysis of hepatic SOD activity in turbot (*Scophthalmus maximus*) under different environmental stresses. (**A**,**B**) Daily fluctuation of SOD activity in the liver. (**A**) Comparison between the control group (green line) and the low-salinity stress group (gray line). (**B**) Comparison between the control group (green line) and the high-temperature stress group (red line). The black bars on the *x*-axis represent the dark phase (night). Asterisks (*) in the line graph indicate significant differences between the treatment group and the control group at the same time point (*p* < 0.05). (**C**–**N**) Cosinor rhythmometry analysis of hepatic SOD activity. The solid blue line represents the best-fit cosine curve, and the shaded area indicates the 95% confidence interval (CI). Red dots represent the observed data points. The *p*-value indicates the significance of the rhythm fit (*p* < 0.05 indicates a significant rhythm). Group assignments: panels (**C**–**F**) represent the control group; panels (**G**–**J**) represent the high-temperature group; and panels (**K**–**N**) represent the low-salinity group. Analysis parameters: (**C**,**G**,**K**) 24 h period (daily rhythm) fitted to 48 h time-series data. (**D**,**H**,**L**) A 24 h period (daily rhythm) fitted to 72 h time-series data. (**E**,**I**,**M**) A 48 h period fitted to 48 h time-series data. (**F**,**J**,**N**) A 72 h period fitted to 72 h time-series data.

**Figure 2:**
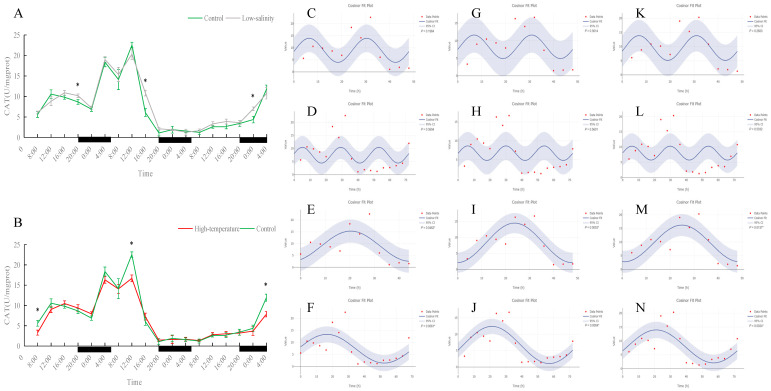
Temporal variations and rhythmicity analysis of hepatic CAT activity in turbot (*Scophthalmus maximus*) under different environmental stresses. (**A**,**B**) Daily fluctuation of CAT activity in the liver. (**A**) Comparison between the control group (green line) and the low-salinity stress group (gray line). (**B**) Comparison between the control group (green line) and the high-temperature stress group (red line). The black bars on the *x*-axis represent the dark phase (night). Asterisks (*) in the line graph indicate significant differences between the treatment group and the control group at the same time point (*p* < 0.05). (**C**–**N**) Cosinor rhythmometry analysis of hepatic CAT activity. The solid blue line represents the best-fit cosine curve, and the shaded area indicates the 95% confidence interval (CI). Red dots represent the observed data points. The *p*-value indicates the significance of the rhythm fit (*p* < 0.05 indicates a significant rhythm). Group assignments: panels (**C**–**F**) represent the control group; panels (**G**–**J**) represent the high-temperature group; and panels (**K**–**N**) represent the low-salinity group. Analysis parameters: (**C**,**G**,**K**) 24 h period (daily rhythm) fitted to 48 h time-series data. (**D**,**H**,**L**) A 24 h period (daily rhythm) fitted to 72 h time-series data. (**E**,**I**,**M**) A 48 h period fitted to 48 h time-series data. (**F**,**J**,**N**) A 72 h period fitted to 72 h time-series data.

**Figure 3:**
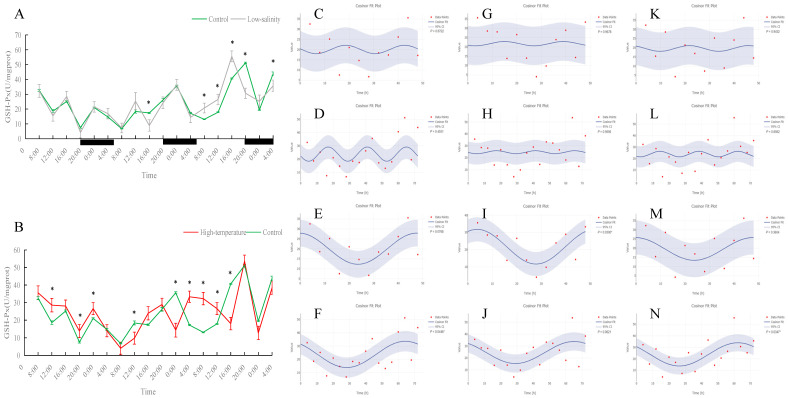
Temporal variations and rhythmicity analysis of hepatic GSH-Px activity in turbot (*Scophthalmus maximus*) under different environmental stresses. (**A**,**B**) Daily fluctuation of GSH-Px activity in the liver. (**A**) Comparison between the control group (green line) and the low-salinity stress group (gray line). (**B**) Comparison between the control group (green line) and the high-temperature stress group (red line). The black bars on the *x*-axis represent the dark phase (night). Asterisks (*) in the line graph indicate significant differences between the treatment group and the control group at the same time point (*p* < 0.05). (**C**–**N**) Cosinor rhythmometry analysis of hepatic GSH-Px activity. The solid blue line represents the best-fit cosine curve, and the shaded area indicates the 95% confidence interval (CI). Red dots represent the observed data points. The *p*-value indicates the significance of the rhythm fit (*p* < 0.05 indicates a significant rhythm). Group assignments: panels (**C**–**F**) represent the control group; panels (**G**–**J**) represent the high-temperature group; and panels (**K**–**N**) represent the low-salinity group. Analysis parameters: (**C**,**G**,**K**) 24 h period (daily rhythm) fitted to 48 h time-series data. (**D**,**H**,**L**) A 24 h period (daily rhythm) fitted to 72 h time-series data. (**E**,**I**,**M**) A 48 h period fitted to 48 h time-series data. (**F**,**J**,**N**) A 72 h period fitted to 72 h time-series data.

**Figure 4:**
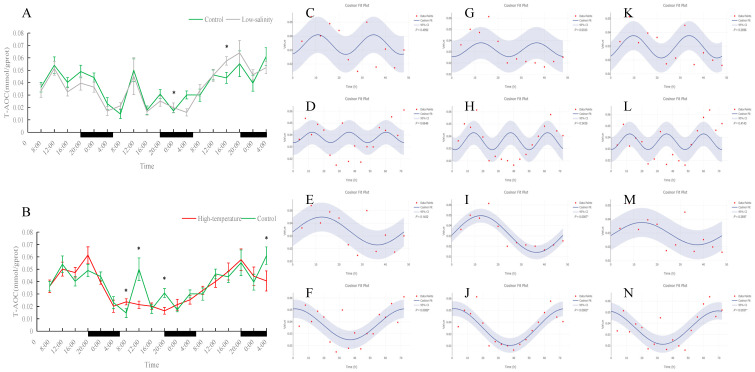
Temporal variations and rhythmicity analysis of hepatic T-AOC content in turbot (*Scophthalmus maximus*) under different environmental stresses. (**A**,**B**) Daily fluctuation of T-AOC content in the liver. (**A**) Comparison between the control group (green line) and the low-salinity stress group (gray line). (**B**) Comparison between the control group (green line) and the high-temperature stress group (red line). The black bars on the *x*-axis represent the dark phase (night). Asterisks (*) in the line graph indicate significant differences between the treatment group and the control group at the same time point (*p* < 0.05). (**C**–**N**) Cosinor rhythmometry analysis of hepatic T-AOC levels. The solid blue line represents the best-fit cosine curve, and the shaded area indicates the 95% confidence interval (CI). Red dots represent the observed data points. The *p*-value indicates the significance of the rhythm fit (*p* < 0.05 indicates a significant rhythm). Group assignments: panels (**C**–**F**) represent the control group; panels (**G**–**J**) represent the high-temperature group; and panels (**K**–**N**) represent the low-salinity group. Analysis parameters: (**C**,**G**,**K**) 24 h period (daily rhythm) fitted to 48 h time-series data. (**D**,**H**,**L**) A 24 h period (daily rhythm) fitted to 72 h time-series data. (**E**,**I**,**M**) A 48 h period fitted to 48 h time-series data. (**F**,**J**,**N**) A 72 h period fitted to 72 h time-series data.

**Figure 5:**
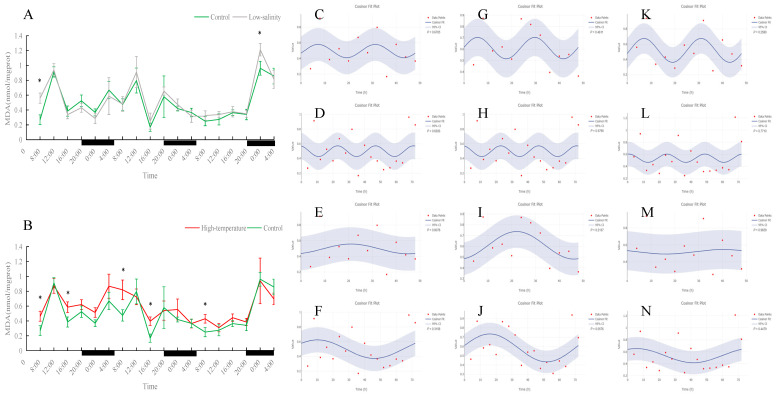
Temporal variations and rhythmicity analysis of hepatic MDA content in turbot (*Scophthalmus maximus*) under different environmental stresses. (**A**,**B**) Daily fluctuation of MDA content in the liver. (**A**) Comparison between the control group (green line) and the low-salinity stress group (gray line). (**B**) Comparison between the control group (green line) and the high-temperature stress group (red line). The black bars on the *x*-axis represent the dark phase (night). Asterisks (*) in the line graph indicate significant differences between the treatment group and the control group at the same time point (*p* < 0.05). (**C**–**N**) Cosinor rhythmometry analysis of hepatic MDA levels. The solid blue line represents the best-fit cosine curve, and the shaded area indicates the 95% confidence interval (CI). Red dots represent the observed data points. The *p*-value indicates the significance of the rhythm fit (*p* < 0.05 indicates a significant rhythm). Group assignments: panels (**C**–**F**) represent the control group; panels (**G**–**J**) represent the high-temperature group; and panels (**K**–**N**) represent the low-salinity group. Analysis parameters: (**C**,**G**,**K**) 24 h period (daily rhythm) fitted to 48 h time-series data. (**D**,**H**,**L**) A 24 h period (daily rhythm) fitted to 72 h time-series data. (**E**,**I**,**M**) A 48 h period fitted to 48 h time-series data. (**F**,**J**,**N**) A 72 h period fitted to 72 h time-series data.

As shown in Figure 1, Figure 2, Figure 3, Figure 4 and Figure 5, under normal, low-salinity, and high-temperature rearing conditions, most antioxidant enzyme indices in juvenile turbot liver did not exhibit significant 24 h rhythms. The exception was SOD under normal conditions. In the control group, SOD activity showed a clear diurnal fluctuation, peaking around midday (*p* < 0.05). This rhythmicity was largely maintained under low-salinity conditions but was disrupted by high-temperature stress.

In contrast, T-AOC, CAT, and GSH-Px showed no significant 24 h cycles in any treatment, although all these indices exhibited significantly longer-term oscillations (at 48 h or 72 h) (*p* < 0.05). Specifically, CAT activity displayed similar 48 h and 72 h cycles in all environments, indicating that neither low salinity nor high temperature significantly altered its fluctuation pattern. T-AOC and GSH-Px also maintained significant 72 h rhythms in each group (*p* < 0.05); the rhythm in the low-salinity group was similar to the control, whereas the high-temperature group exhibited an oscillation pattern different from the control (for example, both T-AOC and GSH-Px showed significant 48 h cycles under high-temperature conditions (*p* < 0.05)), reflecting that the fish developed new rhythmic adaptations to heat stress.

MDA content did not show clear periodic changes in any group (no significant 24 h, 48 h, or 72 h rhythm). The control group’s MDA levels fluctuated somewhat, but the timing of peaks and troughs was irregular; the low-salinity group’s MDA levels and trend were essentially consistent with the control, indicating that long-term low-salinity exposure did not induce significant oxidative damage. In contrast, the high-temperature group had significantly higher MDA levels than the control at most time points (*p* < 0.05), indicating that prolonged heat stress led to continuous accumulation of oxidative damage that the fish could not alleviate in time.

### 3.2. Effects of Different Rearing Environments on Non-Specific Immune Parameters in Juvenile Turbot Serum

**Figure 6:**
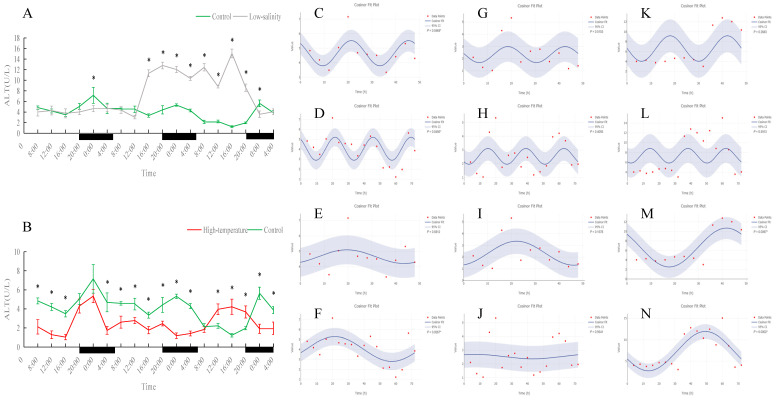
Temporal variations and rhythmicity analysis of serum ALT activity in turbot (*Scophthalmus maximus*) under different environmental stresses. (**A**,**B**) Daily fluctuation of ALT activity in serum. (**A**) Comparison between the control group (green line) and the low-salinity stress group (gray line). (**B**) Comparison between the control group (green line) and the high-temperature stress group (red line). The black bars on the *x*-axis represent the dark phase (night). Asterisks (*) in the line graph indicate significant differences between the treatment group and the control group at the same time point (*p* < 0.05). (**C**–**N**) Cosinor rhythmometry analysis of serum ALT activity. The solid blue line represents the best-fit cosine curve, and the shaded area indicates the 95% confidence interval (CI). Red dots represent the observed data points. The *p*-value indicates the significance of the rhythm fit (*p* < 0.05 indicates a significant rhythm). Group assignments: panels (**C**–**F**) represent the control group; panels (**G**–**J**) represent the high-temperature group; and panels (**K**–**N**) represent the low-salinity group. Analysis parameters: (**C**,**G**,**K**) 24 h period (daily rhythm) fitted to 48 h time-series data. (**D**,**H**,**L**) A 24 h period (daily rhythm) fitted to 72 h time-series data. (**E**,**I**,**M**) A 48 h period fitted to 48 h time-series data. (**F**,**J**,**N**) A 72 h period fitted to 72 h time-series data.

**Figure 7:**
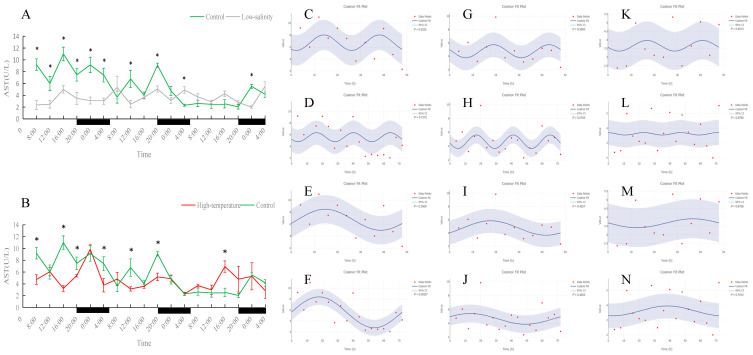
Temporal variations and rhythmicity analysis of serum AST activity in turbot (*Scophthalmus maximus*) under different environmental stresses. (**A**,**B**) Daily fluctuation of AST activity in serum. (**A**) Comparison between the control group (green line) and the low-salinity stress group (gray line). (**B**) Comparison between the control group (green line) and the high-temperature stress group (red line). The black bars on the *x*-axis represent the dark phase (night). Asterisks (*) in the line graph indicate significant differences between the treatment group and the control group at the same time point (*p* < 0.05). (**C**–**N**) Cosinor rhythmometry analysis of serum AST activity. The solid blue line represents the best-fit cosine curve, and the shaded area indicates the 95% confidence interval (CI). Red dots represent the observed data points. The *p*-value indicates the significance of the rhythm fit (*p* < 0.05 indicates a significant rhythm). Group assignments: panels (**C**–**F**) represent the control group; panels (**G**–**J**) represent the high-temperature group; and panels (**K**–**N**) represent the low-salinity group. Analysis parameters: (**C**,**G**,**K**) 24 h period (daily rhythm) fitted to 48 h time-series data. (**D**,**H**,**L**) A 24 h period (daily rhythm) fitted to 72 h time-series data. (**E**,**I**,**M**) A 48 h period fitted to 48 h time-series data. (**F**,**J**,**N**) A 72 h period fitted to 72 h time-series data.

**Figure 8:**
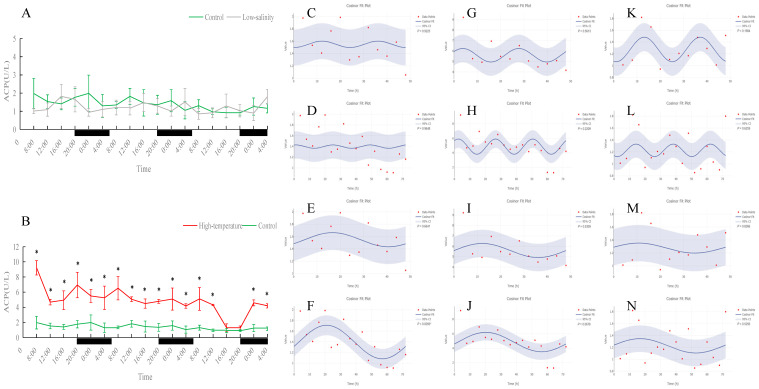
Temporal variations and rhythmicity analysis of serum ACP activity in turbot (*Scophthalmus maximus*) under different environmental stresses. (**A**,**B**) Daily fluctuation of ACP activity in serum. (**A**) Comparison between the control group (green line) and the low-salinity stress group (gray line). (**B**) Comparison between the control group (green line) and the high-temperature stress group (red line). The black bars on the *x*-axis represent the dark phase (night). Asterisks (*) in the line graph indicate significant differences between the treatment group and the control group at the same time point (*p* < 0.05). (**C**–**N**) Cosinor rhythmometry analysis of serum ACP activity. The solid blue line represents the best-fit cosine curve, and the shaded area indicates the 95% confidence interval (CI). Red dots represent the observed data points. The *p*-value indicates the significance of the rhythm fit (*p* < 0.05 indicates a significant rhythm). Group assignments: panels (**C**–**F**) represent the control group; panels (**G**–**J**) represent the high-temperature group; and panels (**K**–**N**) represent the low-salinity group. Analysis parameters: (**C**,**G**,**K**) 24 h period (daily rhythm) fitted to 48 h time-series data. (**D**,**H**,**L**) A 24 h period (daily rhythm) fitted to 72 h time-series data. (**E**,**I**,**M**) A 48 h period fitted to 48 h time-series data. (**F**,**J**,**N**) A 72 h period fitted to 72 h time-series data.

**Figure 9:**
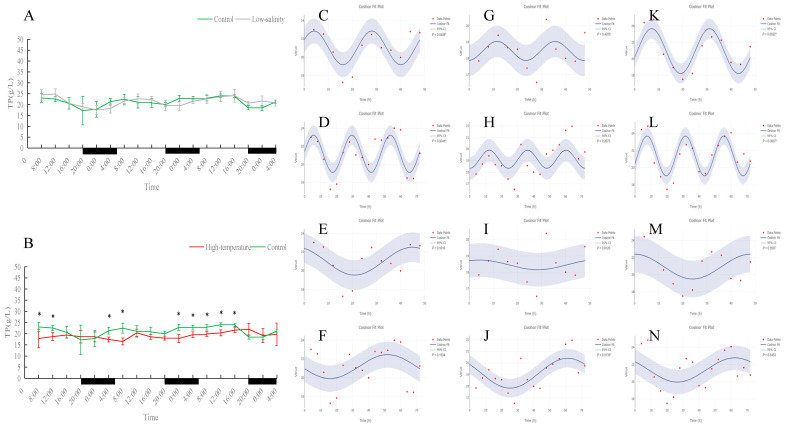
Temporal variations and rhythmicity analysis of serum TP content in turbot (*Scophthalmus maximus*) under different environmental stresses. (**A**,**B**) Daily fluctuation of TP content in serum. (**A**) Comparison between the control group (green line) and the low-salinity stress group (gray line). (**B**) Comparison between the control group (green line) and the high-temperature stress group (red line). The black bars on the *x*-axis represent the dark phase (night). Asterisks (*) in the line graph indicate significant differences between the treatment group and the control group at the same time point (*p* < 0.05). (**C**–**N**) Cosinor rhythmometry analysis of serum TP levels. The solid blue line represents the best-fit cosine curve, and the shaded area indicates the 95% confidence interval (CI). Red dots represent the observed data points. The *p*-value indicates the significance of the rhythm fit (*p* < 0.05 indicates a significant rhythm). Group assignments: panels (**C**–**F**) represent the control group; panels (**G**–**J**) represent the high-temperature group; and panels (**K**–**N**) represent the low-salinity group. Analysis parameters: (**C**,**G**,**K**) 24 h period (daily rhythm) fitted to 48 h time-series data. (**D**,**H**,**L**) A 24 h period (daily rhythm) fitted to 72 h time-series data. (**E**,**I**,**M**) A 48 h period fitted to 48 h time-series data. (**F**,**J**,**N**) A 72 h period fitted to 72 h time-series data.

As shown in Figure 6, Figure 7, Figure 8 and Figure 9, under normal conditions, ALT activity exhibited a significant diurnal rhythm, whereas AST did not show obvious 24 h oscillation. In the control group, ALT activity reached its trough around 16:00 and its peak around 0:00 each day, demonstrating a regular daily cycle. Both low-salinity and high-temperature stress disrupted this ALT rhythm. The low-salinity group, while lacking a 24 h cycle, gradually developed 48 h and 72 h fluctuations; in the high-temperature group, no new orderly rhythm emerged, and ALT activity at most sampling times was significantly lower than in the control (*p* < 0.05), indicating that high temperature caused more severe hepatic functional impairment. AST activity showed only weak rhythmicity in the control (no clear diurnal cycle, only slight fluctuations over the 72 h period). Under low-salinity and high-temperature conditions, AST levels were significantly lower at multiple time points on the first two days compared to the control (*p* < 0.05), further indicating that stress suppressed normal AST fluctuations and adversely affected liver function.

In both the control and low-salinity groups, serum ACP activity remained basically stable throughout the monitoring period, with no significant difference between the groups. In the high-temperature group, ACP activity was significantly higher than the control at almost all time points (*p* < 0.05), indicating tissue damage under heat stress and a state of continuous immune activation. Serum total protein (TP) content showed a clear daily rhythm under normal conditions, which persisted under low-salinity conditions; the TP levels of these two groups remained stable overall with no significant difference during the monitoring period. In the high-temperature group, the TP daily rhythm was disrupted: at most time points, TP content was significantly lower than in the control (*p* < 0.05), and the overall TP level during the monitoring period was noticeably reduced, indicating that prolonged high-temperature stress suppressed the fish’s immune capacity. Notably, although TP levels were overall reduced under heat stress, a new 72 h cyclic fluctuation appeared, suggesting an adaptive rhythmic adjustment by the fish under adverse conditions.

### 3.3. Effects of Different Rearing Environments on Endocrine Parameters in Juvenile Turbot Serum

**Figure 10:**
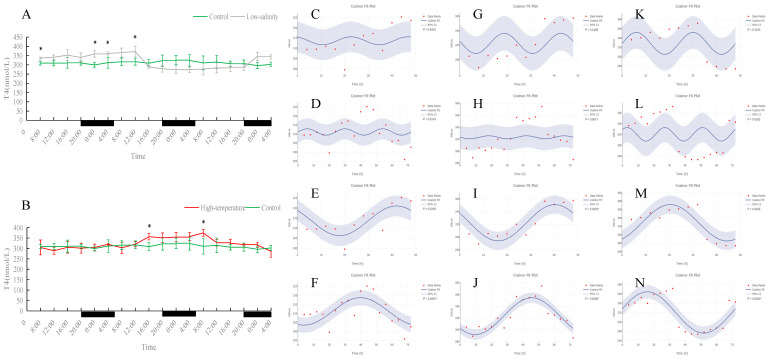
Temporal variations and rhythmicity analysis of serum T4 content in turbot (*Scophthalmus maximus*) under different environmental stresses. (**A**,**B**) Daily fluctuation of T4 content in serum. (**A**) Comparison between the control group (green line) and the low-salinity stress group (gray line). (**B**) Comparison between the control group (green line) and the high-temperature stress group (red line). The black bars on the *x*-axis represent the dark phase (night). Asterisks (*) in the line graph indicate significant differences between the treatment group and the control group at the same time point (*p* < 0.05). (**C**–**N**) Cosinor rhythmometry analysis of serum T4 levels. The solid blue line represents the best-fit cosine curve, and the shaded area indicates the 95% confidence interval (CI). Red dots represent the observed data points. The *p*-value indicates the significance of the rhythm fit (*p* < 0.05 indicates a significant rhythm). Group assignments: panels (**C**–**F**) represent the control group; panels (**G**–**J**) represent the high-temperature group; and panels (**K**–**N**) represent the low-salinity group. Analysis parameters: (**C**,**G**,**K**) 24 h period (daily rhythm) fitted to 48 h time-series data. (**D**,**H**,**L**) A 24 h period (daily rhythm) fitted to 72 h time-series data. (**E**,**I**,**M**) A 48 h period fitted to 48 h time-series data. (**F**,**J**,**N**) A 72 h period fitted to 72 h time-series data.

**Figure 11:**
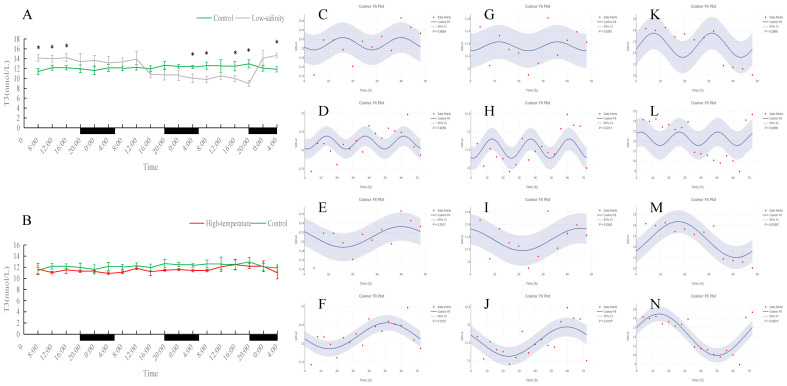
Temporal variations and rhythmicity analysis of serum T3 content in turbot (*Scophthalmus maximus*) under different environmental stresses. (**A**,**B**) Daily fluctuation of T3 content in serum. (**A**) Comparison between the control group (green line) and the low-salinity stress group (gray line). (**B**) Comparison between the control group (green line) and the high-temperature stress group (red line). The black bars on the *x*-axis represent the dark phase (night). Asterisks (*) in the line graph indicate significant differences between the treatment group and the control group at the same time point (*p* < 0.05). (**C**–**N**) Cosinor rhythmometry analysis of serum T3 levels. The solid blue line represents the best-fit cosine curve, and the shaded area indicates the 95% confidence interval (CI). Red dots represent the observed data points. The *p*-value indicates the significance of the rhythm fit (*p* < 0.05 indicates a significant rhythm). Group assignments: panels (**C**–**F**) represent the control group; panels (**G**–**J**) represent the high-temperature group; and panels (**K**–**N**) represent the low-salinity group. Analysis parameters: (**C**,**G**,**K**) 24 h period (daily rhythm) fitted to 48 h time-series data. (**D**,**H**,**L**) A 24 h period (daily rhythm) fitted to 72 h time-series data. (**E**,**I**,**M**) A 48 h period fitted to 48 h time-series data. (**F**,**J**,**N**) A 72 h period fitted to 72 h time-series data.

**Figure 12:**
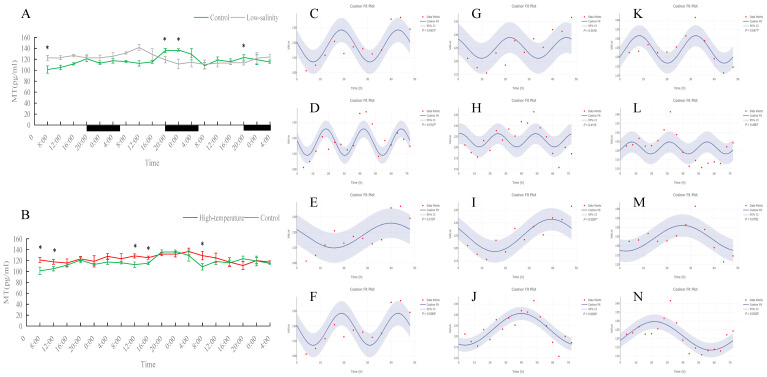
Temporal variations and rhythmicity analysis of serum MT content in turbot (*Scophthalmus maximus*) under different environmental stresses. (**A**,**B**) Daily fluctuation of MT content in serum. (**A**) Comparison between the control group (green line) and the low-salinity stress group (gray line). (**B**) Comparison between the control group (green line) and the high-temperature stress group (red line). The black bars on the *x*-axis represent the dark phase (night). Asterisks (*) in the line graph indicate significant differences between the treatment group and the control group at the same time point (*p* < 0.05). (**C**–**N**) Cosinor rhythmometry analysis of serum MT levels. The solid blue line represents the best-fit cosine curve, and the shaded area indicates the 95% confidence interval (CI). Red dots represent the observed data points. The *p*-value indicates the significance of the rhythm fit (*p* < 0.05 indicates a significant rhythm). Group assignments: panels (**C**–**F**) represent the control group; panels (**G**–**J**) represent the high-temperature group; and panels (**K**–**N**) represent the low-salinity group. Analysis parameters: (**C**,**G**,**K**) 24 h period (daily rhythm) fitted to 48 h time-series data. (**D**,**H**,**L**) A 24 h period (daily rhythm) fitted to 72 h time-series data. (**E**,**I**,**M**) A 48 h period fitted to 48 h time-series data. (**F**,**J**,**N**) A 72 h period fitted to 72 h time-series data.

**Figure 13:**
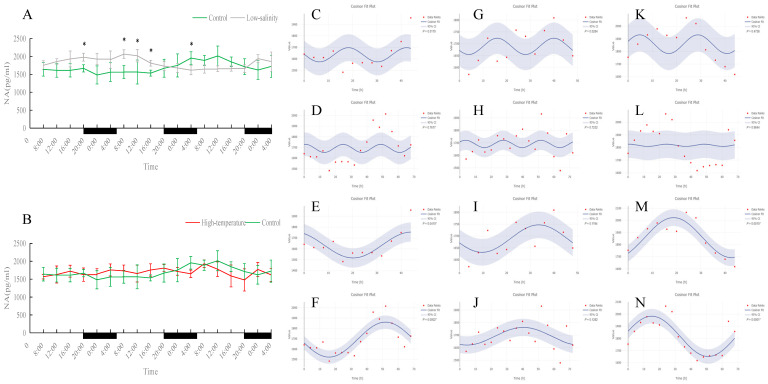
Temporal variations and rhythmicity analysis of serum NA content in turbot (*Scophthalmus maximus*) under different environmental stresses. (**A**,**B**) Daily fluctuation of NA content in serum. (**A**) Comparison between the control group (green line) and the low-salinity stress group (gray line). (**B**) Comparison between the control group (green line) and the high-temperature stress group (red line). The black bars on the *x*-axis represent the dark phase (night). Asterisks (*) in the line graph indicate significant differences between the treatment group and the control group at the same time point (*p* < 0.05). (**C**–**N**) Cosinor rhythmometry analysis of serum NA levels. The solid blue line represents the best-fit cosine curve, and the shaded area indicates the 95% confidence interval (CI). Red dots represent the observed data points. The *p*-value indicates the significance of the rhythm fit (*p* < 0.05 indicates a significant rhythm). Group assignments: panels (**C**–**F**) represent the control group; panels (**G**–**J**) represent the high-temperature group; and panels (**K**–**N**) represent the low-salinity group. Analysis parameters: (**C**,**G**,**K**) 24 h period (daily rhythm) fitted to 48 h time-series data. (**D**,**H**,**L**) A 24 h period (daily rhythm) fitted to 72 h time-series data. (**E**,**I**,**M**) A 48 h period fitted to 48 h time-series data. (**F**,**J**,**N**) A 72 h period fitted to 72 h time-series data.

As shown in Figure 10, Figure 11, Figure 12 and Figure 13, serum T4 and T3 in all groups did not display significant 24 h rhythms but did exhibit clear oscillations on the 48 h and 72 h scales. In the control group, T4 and T3 concentrations remained essentially stable during the 72 h monitoring period. Under prolonged low-salinity stress, T4 and T3 levels fluctuated: initially decreasing and then increasing, resulting in a “stable–decrease–stable–increase” pattern over 72 h; this synchronous fluctuation indicates that under low-salinity stress, T4 and T3 maintained a certain dynamic balance. Under high-temperature stress, T4 concentration also first rose and then fell, whereas T3 concentration remained at low levels throughout the period and was significantly lower than the control at many time points (*p* < 0.05), indicating that high temperature significantly suppressed T3 production. Nevertheless, in both the low-salinity and high-temperature groups, these hormones established new 48 h or 72 h rhythmic cycles, reflecting an adaptive adjustment of endocrine rhythms under stress conditions.

Serum MT levels in the control group displayed clear fluctuations. Under prolonged low-salinity stress, the MT oscillation pattern changed significantly, forming a new oscillatory regime. In the high-temperature group, MT levels were significantly higher than in the control at most time points (*p* < 0.05); although the original diurnal rhythm of MT was disrupted by heat stress, the fish nevertheless established a new cyclic oscillation pattern, indicating an adaptive response. Overall, both stress conditions affected MT rhythmicity, with more pronounced alterations under low-salinity conditions.

Serum NA concentration did not show an obvious 24 h rhythm in any group, but a significant 72 h cycle was observed in both the normal- and low-salinity groups. The NA changes in the low-salinity group were inverse to those of the control (when the control group’s NA decreased, the low-salinity group’s NA increased, and vice versa); nonetheless, the low-salinity group maintained a clear 72 h rhythm, indicating an adaptive adjustment of NA levels under low salinity. In contrast, NA levels in the high-temperature group did not exhibit any regular fluctuations or new periodic rhythm, suggesting that high-temperature stress more severely disrupted NA regulation.

## 4. Discussion

Fish possess an intrinsic circadian clock that generates daily rhythms in most physiological functions [5]. For example, the photoperiod (light/dark cycles) and light intensity directly regulate melatonin secretion and daily rhythms [5]. Other factors—such as water temperature, salinity (osmotic pressure), dissolved oxygen, and stocking density—also influence circadian function. Large deviations in these factors can disturb homeostasis and alter metabolic and behavioral rhythms. Under moderate stress, fish may re-establish a new rhythmic steady state through physiological adjustment, but severe or prolonged stress can collapse existing rhythms, threatening health or survival [31,32]. It is acknowledged that these adjustments occur against the backdrop of fixed feeding schedules, which typically act as synchronizers; however, the distinct rhythmic alterations observed here suggest that environmental stress overrides these feeding cues. Therefore, monitoring circadian changes in physiological indicators can effectively assess environmental adaptability, guide health management, and inform selective breeding for stress resilience. In the present study, although survival was 100% across all groups, the high-temperature group exhibited significantly suppressed growth compared to the control and low-salinity groups. This performance deficit coincided with the observed “rhythmic collapse” in the high-temperature group, whereas the low-salinity fish maintained both “remodeled” rhythms and normal growth. This correlation suggests that the integrity of circadian rhythms is closely linked to production performance. The specific quantitative parameters characterizing these rhythmic patterns are detailed in Table S1.

### 4.1. Effects of Different Farming Environments on Hepatic Antioxidant Enzyme Circadian Rhythms in Turbot

In juvenile turbot of the control group (16 °C, 30 ppt), some liver antioxidant enzymes showed clear day–night oscillations. Notably, SOD activity fluctuated significantly with the light–dark cycle (peaking around midday and troughing near midnight), indicating that under normal conditions, SOD is under clock control and follows a circadian pattern. This finding is consistent with other fish; for example, Ren et al. (2020) reported that SOD activity in the plasma of black sea bass (*Centropristis striata*) exhibited a pronounced circadian rhythm [33]. However, under continuous high-temperature stress (23 °C), this daily SOD rhythm disappeared entirely. While oxidative stress typically induces an antioxidant response initially, the complete loss of rhythmicity here suggests an exhaustion of the antioxidant defense system [34]. The continuous high demand to neutralize ROS may have overridden the circadian regulation, or excessive oxidative stress could have directly impaired the enzymatic function and damaged the molecular clock mechanism [35]. Similar trends have been reported in other species under extreme heat: for instance, in tambaqui (*Colossoma macropomum*) exposed to lethal temperature (37 °C), liver SOD and other antioxidants dropped sharply [36]. In contrast, under moderate low-salinity stress (16 °C, 10 ppt), the SOD rhythm was largely preserved, with peak and trough times similar to the control. This indicates that a moderate salinity decrease did not severely perturb the fish’s circadian antioxidant defense; the fish could maintain a normal SOD rhythm in the low-salt environment.

Other liver antioxidant indices (T-AOC, CAT, GSH-Px) did not show significant 24 h cycles even in control fish. Instead, they oscillated on longer (48–72 h) timescales. Under normal conditions, these multi-day oscillations suggest that their regulation differs from SOD and may be tied to feeding cycles or other longer-term physiological rhythms. In both low-salinity and high-temperature groups, daily rhythms in T-AOC, CAT, and GSH-Px were also absent. However, the long-period oscillation patterns differed between stresses: the low-salinity group maintained a similar 48–72 h oscillation as the control, whereas the high-temperature group showed a marked shift. For example, the main period of T-AOC and GSH-Px changed from 72 h in the control to 48 h in the high-temperature fish. This suggests that under sustained heat stress, the fish generated a new, faster oscillatory rhythm in these defenses, perhaps activating antioxidant responses more frequently to cope with continuous oxidative challenge.

MDA is a marker of lipid peroxidation and overall oxidative stress [37]. In our study, control and low-salinity fish showed no clear periodic changes in hepatic MDA; fluctuations were essentially random, and overall levels were similar between these groups. This suggests that prolonged moderate salinity stress did not cause obvious oxidative damage in turbot—the fish could neutralize any mild ROS increase via their antioxidant enzymes, preventing lipid peroxidation [38]. In contrast, high-temperature stress had a dramatic effect: MDA levels at several time points were significantly higher in the 23 °C group than in controls. This indicates that continuous heat led to excessive ROS accumulation and sustained oxidative stress, overwhelming the antioxidant defenses and causing progressive tissue damage. Similar observations have been reported in other fish: for example, under acute warming, the gills and liver of juvenile tuna significantly increased SOD activity but could not prevent a continued rise in MDA, leading to lipid peroxidation and cellular damage [39].

In summary, high-temperature stress disrupted antioxidant rhythms in juvenile turbot far more severely than low-salinity stress. Heat stress abolished the normal daily rhythms of SOD and other enzymes and caused ongoing oxidative damage, whereas low-salinity stress had relatively minor effects on antioxidant oscillations and allowed the fish to achieve a new steady state.

### 4.2. Effects of Different Farming Environments on Serum Non-Specific Immune Indices in Turbot

ACP is a common indicator of innate immune activity [40]. In control and low-salinity fish, serum ACP activity remained stable throughout the day with no significant differences between groups. This suggests that moderate salinity stress did not elicit a pronounced immune response, and the fish’s innate immune system remained unactivated. By contrast, under high-temperature stress, ACP activity was significantly elevated: the 23 °C group had higher ACP values than controls at almost every sampling time. Sustained elevation of ACP implies that heat stress triggered immune activation [41]. Tissue damage and stress signals likely stimulated macrophage/lysosome activity, releasing more ACP into the blood. Indeed, in fish subjected to acute heat stress, it is common to see a rapid rise in ACP as part of the stress response (followed by complex changes as stress continues) [42,43]. In our study, continuous heat kept ACP high, suggesting a chronic immune-activated or inflammatory state. While long-term innate immune overactivation can be detrimental (draining energy and harming tissues), a short-term ACP increase indicates the fish are actively trying to resist and repair damage caused by high temperature [44,45].

TP reflects overall protein levels, including antibodies, and is a general immune health indicator. Under normal conditions, turbot TP showed a clear daily rhythm (systematically higher at night or day), likely linked to feeding metabolism and circadian patterns of immune protein synthesis and secretion. In the low-salinity group, TP maintained a similar diurnal rhythm to controls, and average TP levels did not differ significantly between these groups. This implies that moderate low-salinity stress did not impair protein synthesis or immune function. In contrast, high-temperature stress had a pronounced effect on TP: the 23 °C group’s daily rhythm was disrupted, and TP levels were significantly lower than controls at most time points, with a lower overall mean. Interestingly, despite this drop, the heat-stressed fish exhibited a new, slower oscillation (about one peak/trough every 72 h). This pattern suggests that high heat caused substantial consumption or suppressed production of immune proteins (e.g., immunoglobulins), lowering serum TP. The fish then may have begun to partially compensate via periodic regulation of protein metabolism. Notably, chronic heat-induced TP decline has been documented in other species; for instance, long-term thermal stress in sturgeons (*Aeromonas hydrophila*) significantly reduced serum ceruloplasmin, lysozyme, and serum amyloid A levels, indicating immune suppression [46]. In our study, the combination of decreased TP and increased ACP under heat stress implies that while the innate immune system was activated (high ACP), the physiological capacity for protein synthesis (including the production of immunoglobulins and other immune-related proteins) may have been compromised.

ALT and AST are key enzymes reflecting liver health [17]. In control fish, ALT activity showed a clear daily rhythm (it was lowest in the evening and peaked around midnight in our trial), likely linked to the diurnal cycle of feeding and metabolism. Similar circadian fluctuations in ALT have been reported in other teleosts, with species-specific timing of daily peaks [47]. Thus, under normal conditions, turbot ALT appears to be clock-regulated and matches the changing liver metabolic demand over the day. By contrast, AST in controls showed no significant diurnal trend and only minor fluctuations, indicating that AST activity is relatively constant compared to ALT—a pattern also seen in other fish species [48]. Under low-salinity stress, turbot ALT lost its 24 h rhythm; the original daily cycle disappeared. However, after extended exposure, the low-salt group showed the emergence of new 48–72 h oscillations in ALT, suggesting that with time, the fish formed a new, slower rhythm under salt stress. Importantly, the magnitude of ALT activity in the low-salinity fish never exceeded that of controls (and was slightly lower at times), indicating that moderate salinity reduction did not cause serious liver damage. In other words, ALT changes under low-salt conditions primarily reflected an adjustment of rhythmic structure. In the high-temperature group, the ALT rhythm disappeared entirely, and ALT activity was significantly lower than in controls at most times. This is unusual because acute liver damage typically causes ALT to spike as the enzyme leaks into the blood. Our finding of reduced ALT under chronic heat may indicate that prolonged stress suppressed liver function: hepatocytes may have been damaged or downregulated so that they produced or released less ALT, or overall metabolism was reduced. Similar declines in multiple enzyme activities (e.g., SOD, CAT, AST) under chronic heat have been reported in other fish, consistent with inhibited hepatic function [49].

In summary, low-salinity stress had a limited impact on turbot immune and liver enzyme indices: aside from a shift in ALT timing, overall ALT and AST levels did not surge, and ACP and TP remained essentially normal. The fish maintained liver function and immune homeostasis under a moderate salinity drop. High-temperature stress, however, caused marked physiological disturbance: liver enzyme rhythms were wiped out, and activities fell (signaling hepatic dysfunction), ACP was persistently high (indicating ongoing inflammation/stress), and TP fell (reflecting weakened humoral immunity). The immunological disruption from heat greatly exceeded that from low salinity, and the circadian disruptions from heat were far more profound.

### 4.3. Effects of Different Farming Environments on Serum Endocrine Hormone Circadian Rhythms in Turbot

Thyroid hormones T3 and T4 play central roles in fish growth and metabolism [50,51]. In our study under normal conditions, neither T3 nor T4 exhibited a clear 24 h rhythm; serum levels remained relatively constant over the day. This matches other reports that in unstressed fish, thyroid hormone secretion is not tightly driven by the daily light–dark cycle but varies with slower factors like temperature or nutrition [52]. In our data, T3 showed a 72 h periodicity, and T4 had both 48 h and 72 h rhythms (likely due to the sampling span). Under low-salinity stress, the rhythmic patterns of T4 and T3 remained essentially the same as controls (though absolute levels and trends differed slightly). Moreover, the changes in T3 and T4 were strongly correlated, suggesting that peripheral conversion from T4 to active T3 stayed consistent. Together, this indicates that moderate low-salinity stress had only a small effect on thyroid hormone secretion. By contrast, under sustained high-temperature stress (23 °C), the overall levels of T3 and T4 remained relatively steady (no sharp diel cycles), but T3 was consistently lower than in controls. This suggests that high heat interfered with T3 synthesis or release, effectively dampening the thyroid axis and slowing metabolism. This outcome aligns with studies in other fish: long-term exposure to elevated temperature often causes a sustained drop in T3 and suppression of thyroid function [53].

The pineal hormone MT is a key output of the circadian clock. In fish, MT is secreted at night and low during the day [5], acting both as a timing signal and as a regulator of metabolism and immunity [54]. In our control turbot, serum MT showed the expected diurnal pattern (high at night, low in the day), indicating normal entrainment by light–dark cycles. Under low-salinity stress, this pattern was dramatically altered: the clear day-night peaks and troughs disappeared, and a new oscillation emerged without obvious day–night contrast. This suggests that osmotic stress induced a neuroendocrine response affecting pineal function, thus re-timing melatonin release. In other words, environmental salinity appears to influence the circadian output of MT, although this phenomenon needs further study. High-temperature stress also had a strong impact on MT. We found that MT levels in the 23 °C fish were significantly higher than controls at many time points—that is, both daytime and nighttime MT were elevated. Nevertheless, the fish did not completely lose MT rhythm; the high-temp group established a new oscillation (e.g., a significant 48 h cycle was detected). Importantly, other studies have noted that fish kept at higher water temperatures often exhibit higher nocturnal MT levels than conspecifics at lower temperatures [55]. Elevated MT under stress is thought to be adaptive: melatonin is a potent antioxidant and an immune modulator. For example, Jung et al. (2016) showed that heat-stressed goldfish (*Carassius auratus*) had high cortisol and reduced immunity, but exogenous MT lowered cortisol and boosted plasma IgM and lysozyme mRNA expression, alleviating heat damage [56]. Thus, the universal rise in MT under heat in our study likely reflects a protective stress response: higher melatonin may help calm the fish, suppress excessive stress hormones, and scavenge free radicals [5,56].

Moreover, we measured NA, a catecholamine involved in the ‘fight-or-flight’ response. Normally, fish NA levels do not exhibit a clear daily rhythm. In control turbot, serum NA fluctuated irregularly over 24 h and only showed a weak 72 h oscillation. Under low-salinity stress, the NA cycle appeared roughly inverted in phase compared to the control but still maintained a pronounced 72 h rhythm. This indicates that salt stress shifted the timing of NA secretion, but the fish preserved a clear periodic pattern by re-phasing its sympathetic output. In stark contrast, high-temperature stress obliterated the NA rhythm entirely. This means heat severely disrupted noradrenergic secretion.

In summary, different stresses had distinct endocrine effects. Low salinity primarily caused a transient shift in thyroid hormones and a phase shift in NA, after which the fish partially restored hormone balance and established new ~72 h cycles. MT rhythm was also altered under low salt, though some oscillation remained. This suggests turbot can adapt to salinity changes by reprogramming their circadian endocrine outputs. By contrast, high-temperature stress induced multi-faceted circadian disruption: the thyroid axis was suppressed (notably lower T3), and daily secretion rhythms were abolished; MT day–night contrast weakened with abnormally high daytime levels; and NA lost any rhythm. Although the fish attempted to adapt by forming new long-period rhythms (e.g., T4 and MT showing 48–72 h cycles), overall high heat caused far more severe circadian dysregulation. These results echo the conclusion that sustained high temperature imposes greater physiological stress on fish than low salinity.

### 4.4. Applications of Circadian Rhythm Changes in Fish Aquaculture

This study systematically compared the daily rhythmic patterns of key physiological indices in juvenile turbot under normal, high-temperature, and low-salinity conditions, revealing how environmental stress remodels fish circadian physiology. In a suitable environment, some antioxidant, immune, and endocrine indicators exhibit clear 24 h rhythms (e.g., SOD, ALT, TP, melatonin), while others oscillate on multi-day cycles or remain relatively constant (e.g., CAT, GSH-Px, T3/T4). Disruptions in these rhythms are closely linked to stress responses, which suggests we can use rhythm changes as biomarkers of fish health or resilience. Monitoring circadian rhythms in fish—especially in antioxidant, immune, and endocrine markers—can therefore provide sensitive health diagnostics. However, we acknowledge that characterizing these cyclic responses typically requires multiple sampling points over time, which inevitably increases laboratory effort and animal usage. To address this in practical aquaculture monitoring, batch sampling strategies can be employed—where small subsets of a cohort are randomly tested to represent the population’s status—thereby avoiding the need to track or stress individual fish repeatedly. For example, superoxide dismutase (SOD) normally shows a distinct daily cycle under healthy conditions; if this daily fluctuation is blunted or lost, it indicates impaired antioxidant defense and emerging oxidative stress. Likewise, ALT and total protein (TP) reflect liver and immune status: changes in the timing or amplitude of ALT oscillation can signal hepatic functional alteration or metabolic disturbance, and reduced TP can indicate immune suppression. Selective breeding for stress tolerance is another promising application. Since traditional rhythm analysis might involve lethal sampling, practical breeding programs can adopt two strategies: prioritizing blood-based markers (e.g., plasma enzymes or hormones) that allow for non-lethal sampling to keep broodstock alive or utilizing genomic selection models, where phenotypic data from sacrificed siblings are used to predict the breeding values of candidates without direct sampling. By choosing individuals that maintain normal physiological rhythms under adverse conditions, we can breed turbot strains with stronger environmental adaptability. In this context, certain indices are especially useful selection traits: AST and ACP. These are biomarkers of liver function and innate immunity, respectively. AST activity (and its circadian fluctuation) reflects the liver’s adaptive capacity and physiological status, while ACP relates to innate immune activation. Under stress (e.g., high temperature), susceptible fish show disrupted AST/ACP patterns and liver suppression. Individuals who keep these markers relatively stable and rhythmic are likely more stress-tolerant. T3 (thyroid hormones): Thyroid hormones are crucial for growth and metabolism. Under stress, sensitive fish typically show large perturbations in T3 levels and loss of normal secretion patterns. Fish that maintain stable T3 homeostasis under changing conditions tend to adapt better to environmental variation. Selecting for such stability could yield strains with broader tolerance.

In conclusion, circadian physiology offers valuable insights and tools for aquaculture. Identifying markers with strong daily rhythms (such as SOD, ALT, and TP) can improve health monitoring. Meanwhile, incorporating circadian traits (e.g., stable AST, ACP, T3, and T4 rhythms under stress) into breeding programs may help develop turbot varieties with enhanced stress resilience.

Limitations of the study: It is important to emphasize that this study focused on biochemical and hormonal outputs—the functional “hands” of the biological clock—rather than the molecular “gears” (e.g., expression of core clock genes Clock, Bmal1, Per, Cry). Therefore, while we observed a functional “collapse” of physiological rhythms under heat stress, future studies at the transcriptional level are required to determine whether this stems from a disruption of the central molecular oscillator or a decoupling of downstream peripheral clocks. In addition, based on our physiological findings, we propose several indicators as prospective candidate biomarkers for screening stress-tolerant strains. However, future studies involving large-scale family selection are needed to quantitatively validate the genetic correlations between these rhythmic traits and long-term survival or growth rates.

## Data Availability

The original contributions presented in this study are included in the article/Supplementary Materials. Further inquiries can be directed to the corresponding author.

## Supplementary Materials

The following supporting information can be downloaded at: https://www.mdpi.com/article/10.3390/antiox15020257/s1, Table S1: Calculated parameters from Cosinor analysis for antioxidant, immune, and endocrine rhythms in turbot under control, high-temperature, and low-salinity regimes.
