# Supplementary material for: Impacts of Long-Term High-Temperature and Low-Salinity Stress on the Circadian Rhythms of Antioxidant, Immune, and Endocrine Systems in Turbot (Scophthalmus maximus)
Source: Antioxidants (Basel). 2026 Feb 17;15(2):257. doi: 10.3390/antiox15020257 (PMC12937773; doi:10.3390/antiox15020257)
Supplement: Supplementary file 1 [file antioxidants-15-00257-s001.zip › antioxidants-4121968-supplementary.pdf]

Table S1. Calculated parameters from Cosinor analysis for antioxidant, immune, and endocrine rhythms in turbot under control, high-temperature, and low-salinity regimes.

	Cosinor Fit	C	D	E	F	G	H	I	J	K	L	M	N
SOD	Mesor	28.6695	26.9741	28.6695	26.9741	31.0174	29.1044	31.0174	29.1044	28.0562	26.8089	28.0562	26.8089
	Amplitude	10.5776	3.9161	5.5158	8.4534	7.4236	1.5029	8.994	6.6015	11.1262	4.5717	3.7321	7.6776
	Acrophase (radians)	0.9554	0.5734	2.8352	1.0606	1.0923	1.5285	-2.7911	1.6288	0.8808	0.6125	2.8607	0.8681
	Acrophase (hours)	3.6493	2.1904	21.6593	12.154	4.1724	5.8384	26.6776	18.6646	3.3644	2.3398	21.8545	9.9473
CAT	Mesor	8.9363	7.4157	8.9363	7.4157	8.2731	6.73	8.2731	6.73	9.5007	8.0127	9.5007	8.0127
	Amplitude	4.9835	2.9977	6.3811	5.9722	3.3624	1.9184	6.2	5.6676	4.3763	2.2748	6.7481	5.9593
	Acrophase (radians)	0.6276	0.3029	2.6654	1.441	0.7512	0.5343	2.5849	1.5059	0.7898	0.4822	2.7386	1.495
	Acrophase (hours)	2.3971	1.1569	20.3623	16.5124	2.8693	2.0409	19.7473	17.2558	3.017	1.8419	20.9216	17.1315
GSH-PX	Mesor	20.094	23.7397	20.094	23.7397	21.7463	24.5919	21.7463	24.5919	19.6158	23.8777	19.6158	23.8777
	Amplitude	2.0538	5.3807	7.8261	9.9318	1.1668	1.2959	10.0854	9.2855	1.5598	2.4123	6.1855	10.1304
	Acrophase (radians)	-2.4681	-2.8941	-0.5783	-1.0206	2.8826	-2.8008	-0.0192	-0.956	-1.6243	2.7132	-0.5554	-1.1931
	Acrophase (hours)	14.5726	12.9455	43.5822	60.3048	11.0109	13.3018	47.853	61.0447	17.7956	10.3638	43.7567	58.3282
MDA	Mesor	0.4944	0.4987	0.4944	0.4987	0.6096	0.5843	0.6096	0.5843	0.5188	0.5355	0.5188	0.5355
	Amplitude	0.0853	0.0734	0.063	0.1266	0.0944	0.0709	0.1279	0.1504	0.1567	0.0687	0.0269	0.1186

	Acrophase (radians)	0.8423	-1.3381	2.2312	0.5419	0.4414	-0.9582	2.3673	1.0438	0.797	-0.8383	-1.5311	0.1374
	Acrophase (hours)	3.2173	18.8889	17.0453	6.2098	1.686	20.34	18.0849	11.9612	3.0444	20.798	36.3029	1.574
T-AOC	Mesor	0.0339	0.0379	0.0339	0.0379	0.0319	0.0359	0.0319	0.0359	0.0296	0.0364	0.0296	0.0364
	Amplitude	0.0071	0.0044	0.011	0.0131	0.0062	0.007	0.0177	0.0155	0.0082	0.0068	0.0082	0.015
	Acrophase (radians)	1.8047	2.1986	1.117	-0.2591	2.2647	2.62	1.1185	-0.2231	1.5249	2.0961	1.2329	-0.5652
	Acrophase (hours)	6.8934	8.3981	8.5331	69.0308	8.6504	10.0076	8.5448	69.4432	5.8246	8.0066	9.419	65.5235
ALT	Mesor	4.6554	4.0509	4.6554	4.0509	2.3333	2.531	2.3333	2.531	6.6126	7.3274	6.6126	7.3274
	Amplitude	0.8678	1.1603	0.4438	1.2497	0.6571	0.5779	1.0299	0.1641	2.569	1.4992	4.0902	4.6012
	Acrophase (radians)	-1.7367	-1.6749	2.0346	1.5256	-2.4743	2.8907	2.3873	0.275	-2.5867	2.6863	-1.3439	-2.3316
	Acrophase (hours)	17.3663	17.6023	15.5431	17.4818	14.5491	11.0415	18.2379	3.1513	14.1197	10.2611	37.7335	45.2815
AST	Mesor	6.7358	5.5687	6.7358	5.5687	4.7533	4.6436	4.7533	4.6436	3.6617	3.6217	3.6617	3.6217
	Amplitude	1.2975	0.7833	1.7578	2.8226	0.9827	0.9816	1.0934	0.7745	0.3106	0.0838	0.2558	0.3315
	Acrophase (radians)	2.6584	-3.0815	1.4089	1.2061	-2.354	-2.8426	1.7985	0.8027	-2.9127	-1.3087	-2.0119	3.0029
	Acrophase (hours)	10.1543	12.2295	10.7631	13.8209	15.0083	13.1419	13.7392	9.1985	12.8742	19.0011	32.63	34.4105

ACP	Mesor	1.549	1.3956	1.549	1.3956	5.5461	4.8492	5.5461	4.8492	1.2746	1.2271	1.2746	1.2271
	Amplitude	0.0519	0.0318	0.1148	0.3142	0.6096	1.0449	0.5938	1.3436	0.2093	0.1041	0.0775	0.1206
	Acrophase (radians)	2.1402	-0.7578	1.6578	1.4823	-0.1969	-0.3775	1.1497	1.4196	2.3881	2.6601	0.8752	1.1147
	Acrophase (hours)	8.1749	21.1055	12.6644	16.986	23.2479	22.558	8.7829	16.2673	9.1217	10.1609	6.6863	12.7731
TP	Mesor	21.0217	21.1763	21.0217	21.1763	18.453	19.1203	18.453	19.1203	20.9433	21.3702	20.9433	21.3702
	Amplitude	1.8496	2.0596	1.4981	1.2854	0.588	0.7808	0.299	1.2864	2.7431	2.3154	1.4505	1.4055
	Acrophase (radians)	0.0243	0.4072	-0.9245	-2.1031	1.9898	2.1465	0.0541	-1.2883	0.8131	0.9198	-0.5097	-1.1844
	Acrophase (hours)	0.0927	1.5555	40.9372	47.9003	7.6004	8.1988	0.4134	57.2373	3.1057	3.5135	44.1059	58.4282
T3	Mesor	12.1015	12.2078	12.1015	12.2078	11.3559	11.5306	11.3559	11.5306	12.677	12.2326	12.677	12.2326
	Amplitude	0.1351	0.1748	0.2248	0.35	0.0725	0.2673	0.1804	0.4108	1.071	0.6793	1.6645	2.265
	Acrophase (radians)	3.1175	2.5178	-1.5637	-2.1626	2.0584	2.4084	-0.8598	-1.4391	0.8463	-0.3953	1.7418	0.8697
	Acrophase (hours)	11.908	9.6174	36.0545	47.2181	7.8625	9.1994	41.4318	55.5097	3.2328	22.4901	13.3064	9.9657
T4	Mesor	313.6745	311.1309	313.6745	311.1309	322.4783	323.225	322.4783	323.225	328.5989	320.3659	328.5989	320.3659
	Amplitude	1.9495	1.7824	7.3966	7.6156	13.9379	2.5611	28.5277	31.3633	23.5842	12.5456	43.4639	44.1984
	Acrophase (radians)	-1.392	0.5218	-1.3238	3.1346	-2.4025	-2.5708	-1.5381	-2.6532	0.5466	-0.2612	2.151	1.0347

	Acrophase (hours)	18.6829	1.9931	37.8873	35.9197	14.823	14.1804	36.2496	41.5971	2.0877	23.0022	16.4326	11.8573
MT	Mesor	117.8711	117.4621	117.8711	117.4621	125.0471	123.3101	125.0471	123.3101	124.174	121.4999	124.174	121.4999
	Amplitude	10.7207	8.4861	8.0857	7.7161	3.9217	3.0737	6.3443	7.3399	7.4553	3.2869	6.9699	8.1424
	Acrophase (radians)	-2.3667	-2.5112	-1.584	-2.7606	-1.6021	-0.7652	-1.6391	3.1406	1.1088	0.8966	2.9561	1.5579
	Acrophase (hours)	14.9599	14.4078	35.8993	40.3654	17.8805	21.0773	35.4784	35.9881	4.2353	3.4247	22.5829	17.8519
NA	Mesor	1,635.40	1,691.11	1,635.40	1,691.11	1,689.18	1,691.24	1,689.18	1,691.24	1,857.13	1,817.24	1,857.13	1,817.24
	Amplitude	61.8033	38.2886	119.3205	169.1681	34.3	29.5498	57.8885	70.3312	74.2071	9.0931	165.9936	166.073
	Acrophase (radians)	-1.4985	0.2302	-0.4973	-1.7564	-3.1413	-0.0795	-2.4061	-3.1042	1.2564	0.1434	2.4577	1.3164
	Acrophase (hours)	18.2761	0.8793	44.2005	51.8727	12.0011	23.6964	29.6187	36.429	4.7989	0.5477	18.7753	15.0853

Note: Group assignments: Panels C-F represent the Control group; Panels G-J represent the High-temperature group; Panels K-N represent the Low-salinity group. Analysis parameters:(C, G, K) 24-h period (daily rhythm) fitted to 48-h time-series data. (D, H, L) 24-h period (daily rhythm) fitted to 72-h time-series data. (E, I, M) 48-h period fitted to 48-h time-series data. (F, J, N) 72-h period fitted to 72-h time-series data.
